# Prevalence of Bovine Genital Campylobacteriosis, Associated Risk Factors and Spatial Distribution in Spanish Beef Cattle Based on Veterinary Laboratory Database Records

**DOI:** 10.3389/fvets.2021.750183

**Published:** 2021-12-08

**Authors:** Nerea Pena-Fernández, David Cano-Terriza, Ignacio García-Bocanegra, Pilar Horcajo, Patricia Vázquez-Arbaizar, Darío Cleofé-Resta, Bárbara Pérez-Arroyo, Luis M. Ortega-Mora, Esther Collantes-Fernández

**Affiliations:** ^1^Servicio Regional de Investigación y Desarrollo Agroalimentario, Villaviciosa, Spain; ^2^Animal Health and Zoonoses Group (SALUVET), Animal Health Department, Faculty of Veterinary Sciences, Complutense University of Madrid, Madrid, Spain; ^3^Animal Health and Zoonosis Research Group (GISAZ), Animal Health Department, Faculty of Veterinary Sciences, University of Cordoba-Agrifood Excellence International Campus (ceiA3), Córdoba, Spain; ^4^Animal Health and Zoonoses Group (SALUVET)-Innova S.L. Faculty of Veterinary Sciences, Complutense University of Madrid, Madrid, Spain

**Keywords:** bovine genital campylobacteriosis, *Campylobacter fetus* subsp. *venerealis*, risk factors, prevalence, spatial distribution, bulls, beef cattle

## Abstract

Bovine genital campylobacteriosis (BGC) is a sexually transmitted disease that causes early reproductive failure in natural breeding cattle that are managed extensively. The aim of this study was to assess the BGC prevalence in Spain from 2011 to 2019 using data collected cross-sectionally from the diagnostic reports issued by the SALUVET veterinary diagnostic laboratory from a total of 5,182 breeding bulls from 1,950 herds managed under “dehesa” systems (large herds within fenced pastures and all-year breeding season) or mountain systems (smaller herds with seasonal breeding management and grazing in communal mountain pastures). Infection was detected by PCR in 7.7 and 12.2% of the bulls and herds tested, respectively. The “dehesa” herd management system (OR = 2.078, *P* = < 0.001, 95% CI = 1.55–1.77), bovine trichomonosis status of the herd (OR = 1.606, *P* = 0.004, 95% CI = 1.15–2.22), and bulls ≥3 years old (OR = 1.392, *P* = 0.04, 95% CI = 1.01–1.92) were identified as risk factors associated with *Campylobacter fetus venerealis* infection. We also studied the high-risk areas for circulation of the infection in extensive beef cattle herds in Spain, showing four significant clusters in “dehesa” areas in the south-western provinces of the country and a fifth cluster located in a mountain area in northern Spain. The results obtained in the present study indicate that BGC is endemic and widely distributed in Spanish beef herds. Specifically, “dehesa” herds are at greater risk for introduction of *Cfv* based on relatively high local prevalence of the infection and the use of specific management practices.

## Introduction

Bovine genital campylobacteriosis (BGC) is a sexually transmitted disease (STD) listed by the World Organization for Animal Health (OIE) and is considered a major cause of early reproductive failure in natural breeding cattle that are managed extensively, placing important restrictions on the international trade of animals and animal products (1, 2). In bulls, the infection is not associated with any clinical signs, and they can become chronic carriers, whereas in cows, the infection is generally self-limiting and can cause embryonic death or early fetal loss (3–7). Herds with BGC often have reduced breeding efficiency, including lower pregnancy rates than expected, an increased number of services per conception, and both extended and longer intervals between calving seasons, resulting in significant economic losses in the affected herds (5, 6, 8–10).

The causative agent of BGC is *Campylobacter fetus* subsp. *venerealis (Cfv)*, a microaerophilic, Gram-negative and motile bacterium with a characteristic spiral form (11). *Cfv* also includes the biotype *intermedius* (*Cfvi*) (12), and both are transmitted during coitus (8, 13). There is another subspecies of *C. fetus* relevant to cattle health: *C. fetus* subsp. *fetus* (*Cff* ), which colonizes the intestine. *Cff* transmission occurs mainly through the fecal-oral route, followed by transient bacteraemia, during which, in pregnant ruminants, the agent can translocate to the placenta, resulting in placentitis and abortion (14, 15). These two subspecies are genetically and phenotypically very similar, which makes their laboratory differentiation difficult (9, 16–18).

BGC is mainly controlled by diagnostic testing, reporting and culling of infected bulls. Antibiotic treatment (streptomycin or oxytetracycline) can be successful in bulls under 3 years old, but it is often not effective in older bulls, thus culling is recommended (1, 19). Unfortunately, and despite several commercial vaccines have been associated with some protection in cattle (9), they are not commercially available in the Europe. BGC has been eradicated in many countries due to the implementation of artificial insemination, especially in dairy cattle (1).

The spatial distribution pattern of BGC correlates with areas where cattle are managed under extensive conditions and natural breeding of cattle is used (5, 9, 20–23). Currently, no studies have attempted to describe the BGC situation in beef cattle herds in Europe and routine diagnostic data from laboratories can be an important source of information. In this work, a retrospective data study, based on 9 years of laboratory test submissions to the SALUVET veterinary diagnostic laboratory (Department of Animal Health, Veterinary Faculty, Madrid, Spain), were applied for *Cfv* detection, among breeding bulls from different areas of Spain. The prevalence of BGC was estimated at animal and herd levels, and potential risk factors for the presence of *Cfv* in Spanish herds were identified. The spatial distribution of the infection in the different Spanish provinces was also analyzed to highlight areas of elevated risk.

## Materials and Methods

### Study Population and Study Design

The targets of this survey were bulls used for natural mating (age ≥15 months) that were subjected to BGC diagnosis by the SALUVET veterinary diagnostic laboratory and originating from farms located in the areas where more than 70% of the Spanish extensive beef cattle population is concentrated (24). Data were collected cross-sectionally from the diagnostic reports issued by SALUVET between January 2011 and December 2019. In order to ensure the representativeness of the study, the required sample size was calculated based on the beef cattle population in these areas (25), an estimated animal and herd prevalence of 13 and 22%, respectively (20), and a 5% accuracy with a 95% confidence level (Win-episcope version 2.0; CLIVE). For the area with the lowest census (data not shown), the sample size required was 22 bulls and 12 herds, whereas for the area with the largest one was 22 bulls and 13 herds.

Samples were analyzed for BGC diagnosis using a PCR as described below. Bulls that tested positive to PCR were considered to be infected with *Cfv* and herds in which at least one bull tested positive for *Cfv* infection were considered positive for BGC. The apparent bull/herd prevalence of *Cfv* infection was established from the proportion of positive bulls/herds to the total number of bulls/herds tested during the study period. Confidence intervals of 95% (95% CI) for proportions were obtained using the exact binomial method. The true prevalence of BGC was estimated based on the sensitivity (97%) and specificity (100%) results previously described (26).

### Sample Collection

SALUVET laboratory provides services for bovine STD diagnosis (BGC and bovine trichomonosis, BT) to private veterinary practitioners. Sampling was performed by preputial scraping of bulls in the field (27) by veterinary practitioners previously trained. Seminars were organized, or a video (https://parasitxpert.es/tecnicas-de-muestreo-para-el-diagnostico-de-la-tricomonosis-ycampilobacteriosis-genital-bovina/) was sent to the veterinarians to demonstrate how to perform preputial scraping to collect smegma samples, as well as to provide specific guidelines for bull sampling (sexual rest of at least 2 weeks) and sample handling (samples kept at 4–37°C, shipped the same or the following day after collection, and received at the laboratory within 24–48 h post-collection) to optimize analytical sensitivity. Collected smegma material was deposited in a 5 ml tube with phosphate-buffered saline (PBS, pH 7.0) and sent to the SALUVET laboratory at room temperature for analysis.

### PCR for *Cfv* Detection

DNA was extracted from a 500 μL aliquot of a mixed PBS sample using the automated DNA extraction system, Maxwell® (Promega, Madison, WI, USA) according to the manufacturer's instructions. For *Cfv* amplification, PCR was performed as previously described (16). Amplified products were visualized under UV light in a 1.8% agarose/ethidium bromide gel. To avoid false-positive reactions, DNA extraction, PCR sample preparation and electrophoresis were each performed in separate rooms with different sets of instruments, and aerosol barrier tips and disposable gloves were used.

The analytical performance of this PCR assay was initially evaluated in our laboratory. The detection limit of *Cfv* by PCR was determined by assaying replicates of serial 10-fold dilutions of *Cfv* DNA, resulting in a detection limit of 26.4 genome copies/μL. The analytic specificity of the assay was verified with a collection of 12 *C. fetus* strains (4 *Cff* and 8 *Cfv*) and 10 non-*fetus Campylobacter* species kindly provided by the Faculty of Veterinary Medicine (Utrecht University, The Netherlands) and the “Laboratorio Central de Veterinaria” (Algete, Madrid, Spain), both Reference Laboratories for Animal Campylobacteriosis, and SALUVET laboratory collection (Supplementary Table 1). All *Cfv* tested in this study were amplified, and *Cff* and the other bacterial species were negative (Supplementary Table 1).

### Data Collection

A dataset was generated in Microsoft® Office Excel for data handling and analysis and included information on the submission from the SALUVET records: identification of the sample or bull, date of receipt of the sample, location of the farm, name of the owner or herd affiliated with the sample, the results of the BGC diagnosis (positive or negative) and BT herd status (negative or positive).

In addition, the veterinarians submitting the samples were required to complete an epidemiologic questionnaire designed to cover potential risk factors for BGC based on the existing literature (22, 28): bull age (≥ or <3 years old), bull breed (native or non-Spanish bull beef breeds), herd size (small ≤ 100 cows or large > 100 cows), bull-cow ratio (≥ or < 1:25), and the presence of reproductive disorders in the herd (yes or no). The farms were also classified according to the type of extensive management system, which in Spain involves two main systems: mountain and “dehesa” systems showing different features (Table 1). Mountain systems are located in central and northern Spain and “dehesa” systems (an agroforestry system comprised of open savannah-like evergreen oak -*Quercus* spp.- woodlands and a typical landscape with a mosaic of croplands, grasslands and scrublands) are located in central, western and southern Spain (29, 30). In order to encourage veterinarians to respond to the questionnaire, some of the participants were also contacted by telephone or email.

**Table 1:** Main features of the Spanish extensive management cattle systems included in this study.

	**Mountain system farms**	**Dehesa system farms**
**Geographical location**	Mountain areas from central and northern Spain	Lowland areas from western and southern Spain
**Climate**	-Mountain	-Mediterranean-continental
	-Oceanic	-Mediterranean
**Average herd size**	45 cows	190 cows
**Main cattle breeds for breeding**	Native breeds: Alpine Brown, Asturiana de la Montaña, Asturiana de los Valles, Rubia gallega	-Native breeds: Retinta, Avileña, Morucha -Non-Spanish breeds: Charolais and Limousin -Crossbreeds
**Grazing areas**	Fenced pastures	Not fenced communal pastures (seasonal movements of cattle from the valley areas near the farms to the high mountain grazing pasture lands in late spring-summer)
**Reproductive practices**	-Restricted breeding season (late spring- summer or late winter-spring) -Communal bulls	-Continuous breeding season (mostly) -Seasonal breeding season (late winter-spring)

Diagnostic testing and data collection were performed from January 2011 to December 2019, whereas database generation and data analysis were conducted after 2019.

### Statistical Analyses

Associations between the presence of *Cfv* infection (dependent variable) and the selected explanatory variables, both at herd-level (type of management, herd size, bull-to-cow ratio, BT herd status and reproductive disorders in the herd) or at animal-level (bull age, bull breed and date of sampling) were initially screened using Pearson's chi-square or Fisher's tests, as appropriate. Variables with *P*-values < 0.20 in the bivariate analysis were further scrutinized for associations using Spearman's rank correlation coefficient (*r*) to avoid collinearity problems. When collinearity (*P* < 0.05 and *r* > 0.4) occurred, only the variable more clearly linked to *Cfv* infection was retained. The next step involved a multiple logistic-regression model using a non-automatic backward selection of variables. Two independent models were constructed to determine the risk factors, one at herd-level and one at animal-level. A forward introduction of variables was used, starting with the variable with the lowest *P*-value in bivariate analysis. At each step, the confounding effect of the included variable was assessed by computing the change in the odds ratios (OR). Changes in the OR >30% were considered indicative of confounding. The model was rerun until all remaining variables presented statistically significant values (likelihood-ratio Wald's test, *P* < 0.05) and a potential relationship with the response variable existed. The fit of the models was assessed using a goodness-of-fit test (31).

A spatial statistical scan was carried out to detect significant clusters (*P* < 0.05) using a Bernoulli model (32). The number of Monte Carlo simulations was set at 999 for the cluster scan statistic. The analysis was performed using SaTScanTM, v9.6.

This study was done and reported in accordance with the Strengthening the Reporting of Observational Studies in Epidemiology (STROBE) checklist (33).

## Results

### Descriptive Data and Questionnaire Response

**Figure 1:**
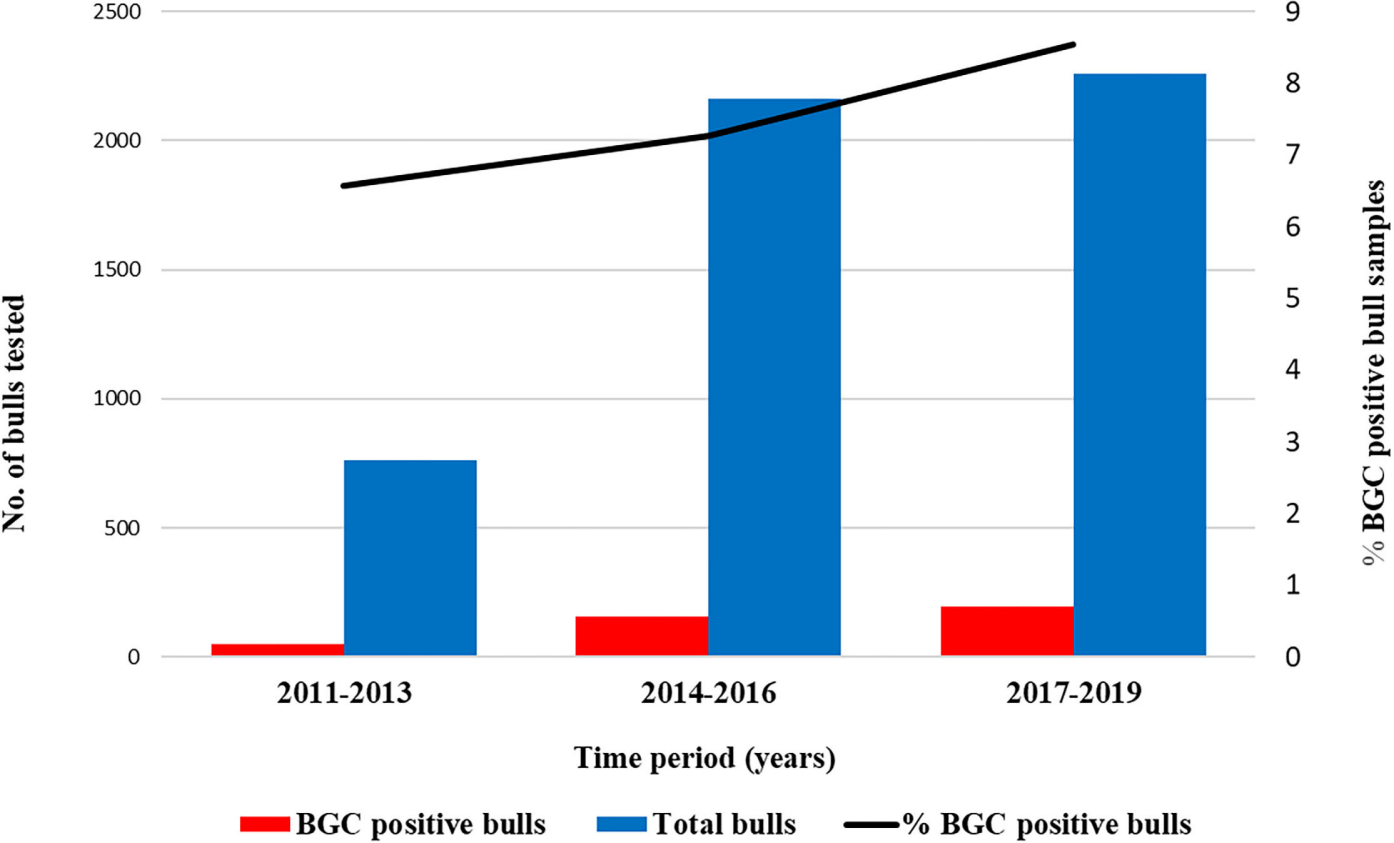
Number of samples analyzed by the SALUVET diagnostic laboratory and apparent prevalence of the bulls and herds examined in the studied period 2011–2019. Data were grouped into 3-year periods to facilitate the observation of testing patterns.

A total of 5,182 breeding bulls were analyzed between January 2011 and December 2019. Bulls younger than 15 months old and bulls from artificial insemination centers were discarded. The number of bulls tested increased from 2011 to 2013 (mean 256.3 bulls/year, ± 58.5) to 2017–2019 (mean 753.3 bulls/year, ± 57.5) (Figure 1). Data about the location and BT status were available for all bulls, and owner information was available for 87.3% of animals. Therefore, it could be established that animals were managed in 1,950 herds with a mean of 2.3 (± 2.3) bull samples per farm. In addition, ~60% of the surveys provided bull breeds and age information, and 30–44% reported data about herd size and the presence or absence of reproductive disorders in the herd.

The bulls and herds analyzed in this study originated from 33 out of 50 Spanish provinces. The number of bulls and herds sampled was representative, corresponding to 5.5% (5182/93928) and 4.1% (1950/46761) of bull and herd population from these areas, respectively. The sampled bulls had a mean age of 4.2 (± 2.1) years old, with 74.9% of the bulls ≥ 3 years of age. There were 23 different breeds represented in the survey (Table 2), with Limousin being the most common imported breed (944/1730; 54.6%) and Pyrenean brown being the most frequent native breed (582/1412; 41.2%). The bulls in the survey were from herds with a mean herd size of 133.3 (± 148.3) cows, and the mean bull-to-cow ratio per herd was 1:36.5 (± 19.60). In addition, 62.5% (3241/5182) of the bulls and 76.3% (1488/1950) of the herds sampled came from mountain management systems whereas 37.5% (1941/5182) of the bulls and 23.7% (462/1950) of the remaining herds came from “dehesa” management systems. A total of 45.7% (387/847) and 17.4% (339/1950) of the herds submitting bulls reported the presence of reproductive problems and positive BT status in the herd, respectively.

**Table 2:** Results of the bivariate analysis of potential risk factors associated with *Campylobacter fetus* subsp. *venerealis* infection in breeding bulls and extensive beef cattle herds in Spain.

**Variable**		**Category**	***Cfv*** **status**			***P*-value**
			**N^**a**^**	**Infected (%)**	**Non-infected (%)**	
Bull level	Bull age^b^	<3 years old	748	49 (6.6)	699 (93.4)	**0.025**
		≥3 years old	2,239	199 (8.9)	2,040 (91.1)	
	Bull breed^c^,	Native	1,411	137 (9.7)	1,274 (90.3)	0.119
		Non-Spanish	1,731	146 (8.4)	1,585 (91.6)	
	Period of sampling	2011–2013	761	50 (6.6)	711 (93.4)	0.124
		2014–2016	2,161	157 (7.3)	2,004 (92.7)	
		2017–2019	2,260	193 (8.5)	2,067 (91.5)	
Herd level	Type of herd management^d^	Mountain	1,488	149 (10)	1,339 (90)	**<** **0.001**
		“Dehesa”	462	87 (18.8)	375 (81.2)	
	Bull-to-cow ratio in the herd^e^	<1:25	151	22 (14.6)	129 (85.4)	0.484
		≥1:25	474	72 (15.2)	402 (84.8)	
	Herd size (No. cows)^f^	≤ 100	360	26 (10)	334 (90)	**<** **0.001**
		>100	260	22 (21.2)	238 (78.8)	
	BT herd status^g^	Positive	339	57 (16.8)	282 (83.2)	**0.003**
		Negative	1,611	179 (11.1)	1,432 (88.9)	
	Reproductive disorders in the herd^h^	Yes	387	50 (12.9)	337 (87.1)	0.269
		No	460	52 (11.3)	408 (88.7)	

*N, number of examined bulls and herds*.

a *Missing values omitted*.

b *Bull age data were recorded for 2,987 bulls out of 5,182 bulls tested*.

c *Bull breed data were recorded for 3,142 bulls out of 5,182 bulls tested. The native breeds documented were “Parda pirenaica” (n = 582), “Asturiana de los valles” (n = 375), “Asturiana de la montaña” (n = 252), “Lidia” (n = 64), “Retinta” (n = 59), “Avileña” (n = 54), “Bruna de los Pirineos” (n = 9), “Berrendo en colorado” (n = 7), “Morucha” (n = 6), “Blanca cacereña” (n = 3) and “Rubia Gallega” (n = 1). The non-Spanish breeds documented were Limousin (n = 944), Charolais (n = 617), Blonde d'Aquitaine (n = 57), Brown Swiss (n = 25), Aberdeen angus (n = 19), Salers (n = 18), Fleckvieh (n = 15), Holstein (n = 15), Belgian blue (n = 13), Black angus (n = 3), Aubrac (n = 2) and Wagyu (n = 2)*.

d *Out of the 1,950 herds analyzed 1,488 belonged to mountain systems and 462 belonged to “dehesa systems*.

e *Bull-to-cow ratio data were recorded for 625 herds out of 1,950 herds tested*.

f *Herd size data were recorded for 620 herds out of 1,950 herds tested*.

g *Herd BT status data were recorded for 1,950 herds out of 1,950 herds tested*.

h *Reproductive disorders in herd data were recorded for 847 herds out of 1,950 herds tested*.

*The bold value means the results that showed significant differences in the bivariate analysis*.

### *Cfv* Prevalence and Associated Risk Factors

The apparent prevalence of the bulls and herds examined in the studied period was 7.7% (400/5182; 95% CI: 7.0–8.4%) and 12.2% (238/1950; 95% CI: 10.8–13.7%), respectively. No significant time-dependent increase (*P* = 0.124) was observed in the bull prevalence, ranging from 6.6% (50/761) in 211–2013 to 8.5% (193/2260) in 2017–2019 (Figure 1; Table 2). The bull and herd estimated true prevalence were 7.9 and 12.6%, respectively.

The results obtained from bivariate analysis (Table 2) showed that *Cfv* infection was found significantly more often in bulls ≥ 3 years (*P* < 0.05), herds from the “dehesa” system (*P* < 0.001), herds with more than 100 cows (*P* < 0.001) and herds with positive BT status (*P* < 0.05). In the multivariate analysis, the variable “bull breed” was excluded due to collinearity with the variable “type of management” (*P* < 0.001, *r* = −0.525). The final multiple logistic-regression model (Table 3) showed that the age was the main risk factor associated with *Cfv* infection in bulls at animal-level, whereas the management system and BT status were identified as the main risk factors at herd-level. Significant higher rates of *Cfv* infection were found in herds belonged to “dehesa” system than in those from mountain system (OR = 2.078, *P* = < 0.001, 95% CI = 1.55–2.77) and in herds with a BT-positive status (OR = 1.606, *P* = 0.004, 95% CI = 1.15–2.22). *Cfv* infection rates were significantly higher in bulls ≥ 3 years old than in animals < 3 years old (OR = 1.392, *P* = 0.046, 95% CI = 1.00–1.92).

**Table 3:** Results of the logistic regression analysis of potential risk factors associated with *Campylobacter fetus* subsp. *venerealis* in breeding bulls and extensive beef cattle herds in Spain.

**Variable**		**Category**	***P*-value**	OR 95%CI*
Bull level	Bull age	<3 years old	_a_	_a_
		≥3 years old	0.046	1.392 (1.006–1.924)
Herd level	Type of herd management	Mountain	_a_	_a_
		“Dehesa”	<0.001	2.078 (1.556–2.774)
	BT herd status	Negative	_a_	_a_
		Positive	0.004	1.606 (1.158–2.227)

* *OR, Odds ratio; 95% CI, 95% Confidence interval*.

a *Reference category*.

### Spatial Distribution

**Figure 2:**
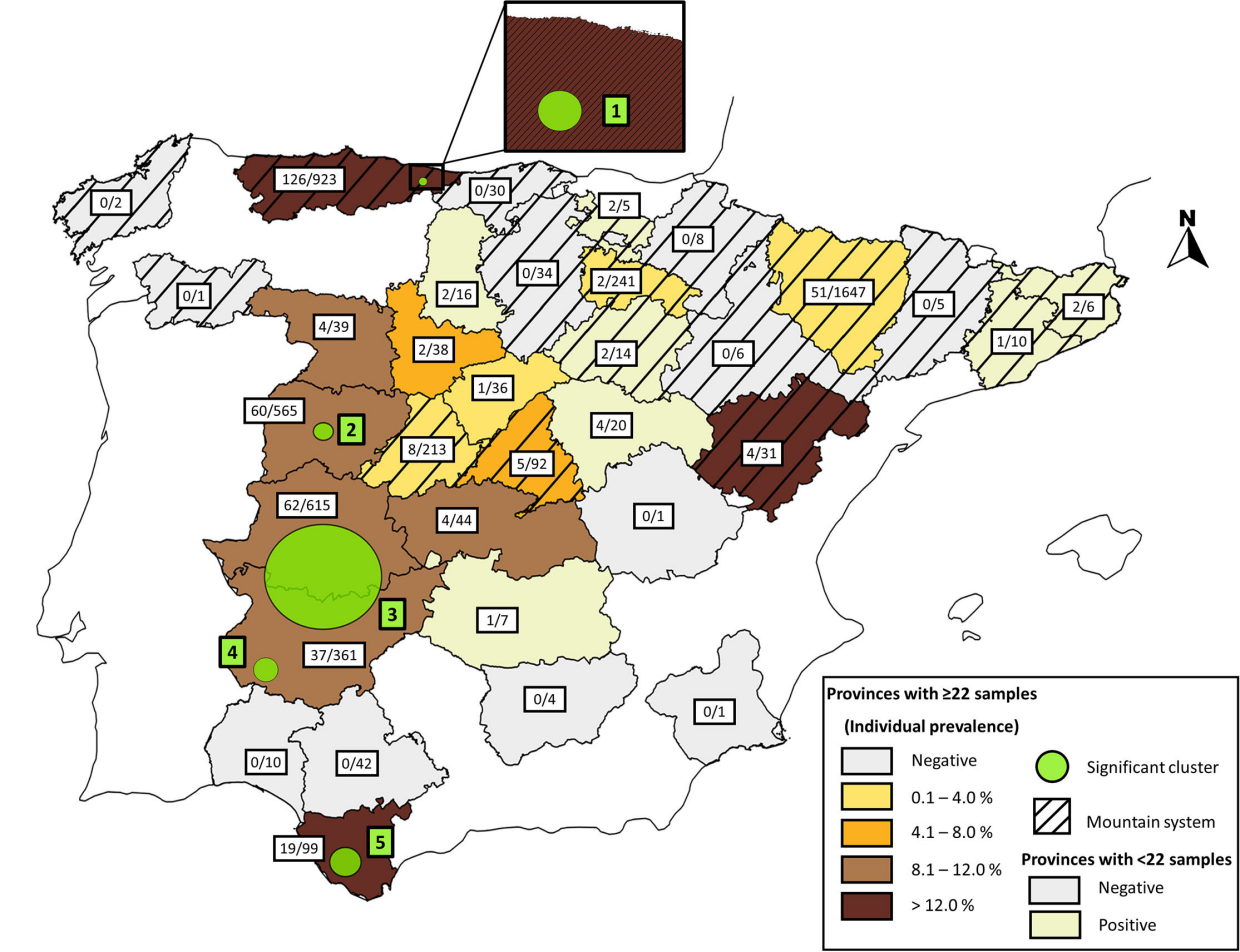
Map of Spain overlaying the areas with a potential risk of BGC represented with a circle and the apparent prevalence of *Cfv* in bulls between 2011 and 2019, colored according to their percentage value. The striped and unlined patterns indicate mountain and “dehesa” areas, respectively.

The distribution of *Cfv*-positive bulls in the different Spanish provinces is shown in Figure 2. From the 17 provinces with representative sampling (≥ 22 bulls), 10 (58.8%) provinces had at least 4% positive cases within the last 9 years, while seven (41.2%) provinces had <4% cases in the same timeframe. The spatial analysis identified five statistically significant clusters of high *Cfv* prevalence (Table 4; Figure 2). The first cluster was located in the Principado de Asturias province (northern Spain), which is representative of mountain systems. Clusters 2, 3, 4, and 5 belonged to the “dehesa” areas and were located in the provinces of Salamanca, Badajoz, Cáceres (western Spain) and Cádiz (southern Spain), respectively.

**Table 4:** Results of the spatial analysis for *Campylobacter fetus* subsp. *venerealis* in breeding bulls from extensive beef herds in Spain.

**Clusters**	**Province**	**Population**	**Cases**	**Expected cases**	**Cases (%)**	**RR^**a**^**	***P*-value**
1	Principado de Asturias	169	48	13.97	28.4	3.81	<0.001
2	Salamanca	45	13	3.72	28.9	3.59	0.036
3	Cáceres	147	34	12.15	23.1	2.98	<0.001
4	Badajoz	29	17	2.40	58.6	7.39	<0.001
5	Cádiz	54	19	4.46	35.2	4.44	<0.001

a *RR, Relative risk*.

## Discussion

BCG is a widespread disease worldwide and is linked to the beef cattle sector, where natural mating is used and cattle are extensively managed such as in western North America, Australia, Africa and Latin America (1, 4, 5, 21, 34). In the last 10 years, the OIE has reported the presence of BGC in several European countries including Ireland, The United Kingdom, France, Germany, Poland and Spain (35). However, the presence of BGC depends on the self-reporting of the country, and this does not provide reliable data. Consequently, the presence of BGC could be underestimated, and the disease may be more widespread on this continent, especially in countries with free-ranging extensive beef breeding systems using natural services. We recently showed that BT (another STD studied together with BGC because the ecology, epidemiology, clinical signs and lesions of both are similar) remains endemic in breeding bulls from Spanish beef herds (27, 36). In the present study, using data from laboratory test submissions to the Animal Health Laboratory (SALUVET), we assessed the bull and herd prevalence and spatial distribution of *Cfv* infection in breeding bulls from Spanish beef herds. Overall, *Cfv* was detected in 7.7% bulls and 12.2% herds over the studied period. Our data also showed that BGC was present in different areas and management systems in Spain, over a period of 9 years (2011–2019), showing an endemic character, since the number of positive breeding bulls remained fairly constant throughout the study period. Information about the situation of BGC in Europe is very limited. Analysis of the veterinary laboratory data provides a potential opportunity for BGC monitoring at the national scale, but the information should be interpreted cautiously, since there are always concerns over how well the sample population reflects the true population, because of the lack of randomization of the analyzed samples. This is due to the increased likelihood that herds from which samples are submitted for testing could be herds where there is already a higher suspicion of infection (herd with reproductive disorders), which could result in overestimating BGC prevalence. However, more than the half (54.3%) of the herds submitting bulls showed no indication of reproductive problems in the herd of origin. Consequently, we consider that the error related to the voluntary participation in this sampling would be almost negligible. Moreover, although available BGC prevalence data are scarce in the literature, our data are also consistent with estimates from previous cross-sectional studies from Argentina, Nigeria or Tanzania, where the bull prevalence ranged from 2.4 to 13.3% (20, 37, 38).

BGC is a moderately to highly transmissible infection, and certain management factors have been shown to contribute to the introduction of the agent into the herd and cattle-to-cattle transmission, particularly those related to animal movement, pasture management, or biosecurity measures (1). In the present study, the use of bulls ≥ 3 years was identified in the multivariate analysis as a potential risk factor. It is widely known that older bulls are more likely to be infected, acting as chronic carriers of causative agents of venereal infections (3, 19, 20, 39, 40), due to a higher number of sexual contacts during their life than younger bulls and to social dominance, since older bulls breed a larger number of cows, increasing the risk of infection by *Cfv*. This is also related to the presence of more folds and deeper glandular crypts in the preputial and penile epithelium of the older bulls, which would promote the creation of an environment conducive to the growth and chronification of *Cfv* infection. The BT herd status was also a risk factor for BGC, which could be related with the mode of transmission and the biological characteristics of *Tritrichomonas fetus* and *Cfv* (22, 28). The third potential risk factor identified for *Cfv* infection was the type of management system. Specifically, BGC was more often diagnosed in bulls from the “dehesa” systems compared to those raised in mountain systems. Particular herd management practices in “dehesa” herds could increase the likelihood of *Cfv* transmission. In most farms managed under “dehesa” systems, breeding season is continuous which is a specific risk factor for STD (22, 41). This contrast with mountain system farms where breeding is seasonal and thus, bulls are separated from cows, stopping the reinfection (42). “Dehesa” herds were also significantly larger than those described in mountain systems. In this regard, more interactions among animals in larger herds has been described, which could promote pathogen transmission (20, 21, 41). In fact, the association between *Cfv* infection and larger herds was initially revealed in the bivariate analysis. Risk of infection could be also higher in large herds with a greater number of bulls, since cow mating can occur with two or more bulls (43, 44). In addition, this risk of acquiring the infection is higher in “dehesa” systems where the rate of external replacement is high and the entry of new bulls for crossbreeding purposes is likely. Additional studies are necessary to investigate particular “dehesa” management practices to control the spread of *Cfv* infection. In addition, it has been suggested that the environmental temperature could increase the bacterial load of *Cfv* in bulls by causing changes in the temperature in the preputial cavity (45). This hypothesis could explain why *Cfv* was more commonly found in geographical areas with relatively high temperatures, such as “dehesa” areas from our country, characterized by a Mediterranean climate. On the other hand, BGC was not associated with reproductive disorders in Spanish herds. BGC is an important cause of early reproductive failure in cattle, resulting in lower pregnancy rates and severe economic losses (46). Additional studies are necessary to estimate the impact of BGC.

BGC was widespread in Spain and was detected in 12.2 and 63.6% of the herds and provinces tested, respectively. We also carried out a spatial analysis to identify high-risk areas for BGC circulation in extensive beef cattle herds in Spain. Data from this 9-year period revealed five spatial clusters that were significantly more likely to contain bulls infected with *Cfv*, than animals located in other areas. Four of these clusters were located in “dehesa” western-south provinces of the country. These high-risk provinces are major beef cattle-producing areas of Spain (24). This finding supports the hypothesis of local transmission of the infection between neighboring herds from these high-risk areas, due to a shared contact network (i.e., between-herd contacts and local cattle movements). Consequently, “dehesa” herds are at greater risk for introduction of *Cfv* based on relatively high local prevalence of the infection and the use of management practices that could increase the risk of the introduction of BGC into the herd. This information can be useful for farmers and veterinarians to make more informed decisions about managing biosecurity risks through animal trade or local contacts, since in Spain, STD control remains strictly voluntary. On the other hand, one cluster was located in a north mountain area located in Principado de Asturias province. A previous epidemiological survey of BT and BGC in the same province showed a high bull prevalence of BT (30%), but *Cfv* was not detected by either culture or PCR (27). Clustering of BGC in this area may have occurred because infected bulls could have shared communal pastures with other herds in this area. Moreover, the use of communal bulls is also a frequent breeding practice in mountain farms which increases the risk of STD transmission (27). This result may suggest that factors directly related to management herd practices and probably not the geographic area are involved. More epidemiological studies are needed to explain the variation in venereal disease presence in Spain.

In terms of diagnosis, bulls should be the target for diagnostic investigations, epidemiological studies, as well as control and prevention strategies (9). Accordingly, accurate diagnosis is necessary to avoid the spread of BGC and consequent economic losses. The laboratory diagnosis of *Cfv* by microbial culture, direct immunofluorescence test and PCR can be problematic due to different factors related to its growth and the differentiation between *Cfv* and *Cff* (9, 45, 47, 48). Bacteriologic culture with subsequent phenotypic identification remains the gold standard for the detection of *Cfv* (2). Nevertheless, isolation of *Cfv* from field samples is difficult, showing low sensitivity (9, 45, 47, 48). Subsequently, the majority of *Cfv* detection is currently achieved by PCR assays. In the present study, PCR targeting the insertion element ISC*fe*1 was used for the detection of *Cfv* directly from preputial samples, which permits the identification of *Cfv* (16). This PCR assay has shown a high sensitivity (97%) and specificity (100%) (26, 49). However, a previous study demonstrated that ISC*fe*1 element-based PCR was associated with specificity failures, and validation of positive results is advisable by confirmatory tests to discriminate true- from false-positive results (50). Here, verification of some PCR-positive results was carried out through repeated sampling and bacteriologic culture from PCR-positive bulls (data not shown). Accordingly, our data on repeatability, coupled with validation in our laboratory and published data, indicate that the diagnostic method used in our study and the results obtained in the present study can be considered reliable. In previous studies, PCR specificity problems for BGC diagnosis have been suggested to be related to the presence of cross-reactions with *Cff*, the preputial saprophytic flora or to the genetic variability of *Cfv* isolates from different geographical areas (51, 52). In Spain, there are also some concerns about *Cfv*-positive results for virgin or young bulls given by some commercial laboratories (personnel communication). A previous study also reported that the specificity of PCR targeting the *parA* gene (53) was only 85% in virgin bulls (45). Diagnostic laboratories should focus on minimizing false results and, in particular, for BGC diagnosis in detecting false-positive results from non-specific PCR reactions, which is currently an important concern. False positive/negative results can cause economic losses due to the costs of bull testing, culling of valuable bulls, and other potential control measures such as antibiotic treatment or herd vaccination, in countries where commercial vaccines are licensed (not in Europe). More investigation into possible sources of PCR cross-reaction will help to improve BGC diagnosis.

## Conclusions

The results obtained indicate that BGC is endemic and widely distributed in Spanish beef herds occurring more commonly in bulls ≥ 3 years old, in herds with a BT-positive status and was spatially clustered in herds from “dehesa” systems, located in the west and southwest of the country. Moreover, this study also identified some specific mountain areas in Spain with an increased BGC risk, where the use of communal pastures and communal bulls are frequent. Thus, it is recommended the use of young bulls and systematic testing of breeding bulls should be part of the prevention and control programmes for STD in Spanish beef herds. The identification of spatial clusters of BGC in this study can also serve to inform risk-based, more cost-effective strategies toward better prevention and control of this STD in Spain. Awareness of the economic importance of this sector in Spain and due to the capacity of BGC to cause great economic losses, collective action must be established to implement further measures for the prevention and control of BGC. Moreover, the importance of STD (BGC and BT) could even increase in the future, due to the current trend (especially in European western countries) toward more extensive and traditional farming systems. In view of our results, more studies should be undertaken to investigate the situation of bovine STD in other European countries where natural breeding of beef cattle is common and there are no monitoring programmes. We also propose the adoption of validated and uniform tests for BGC diagnosis to ensure that results are fully equivalent among diagnostic laboratories.

## Data Availability Statement

The raw data supporting the conclusions of this article will be made available by the authors, without undue reservation.

## Author Contributions

LO-M and EC-F conceived the study and participated in its design. PH, NP-F, and PV-A performed the sample analysis and the PCR tests. NP-F, EC-F, PV-A, DC-R, and BP-A prepared the database. DC-T and IG-B carried out statistical analyses and interpreted the results. NP-F and EC-F wrote the manuscript, with interpretation of results, material and methods and discussion inputs from DC-T, IG-B, PH, and LO-M. All authors contributed to the article and approved the submitted version.

## Funding

This study was supported by several research projects (RTA2017-00076-00-00 and PLATESA2-CM P2018/BAA-4370) and the grant for doctoral formation (Pre2018-086113 funded by MCIN/AEI/10.13039/501100011033 and FSE) awarded to PhD student NP-F.

## Conflict of Interest

The authors declare that the research was conducted in the absence of any commercial or financial relationships that could be construed as a potential conflict of interest.

## Publisher's Note

All claims expressed in this article are solely those of the authors and do not necessarily represent those of their affiliated organizations, or those of the publisher, the editors and the reviewers. Any product that may be evaluated in this article, or claim that may be made by its manufacturer, is not guaranteed or endorsed by the publisher.
