# Supplementary material for: Prevalence of Bovine Genital Campylobacteriosis, Associated Risk Factors and Spatial Distribution in Spanish Beef Cattle Based on Veterinary Laboratory Database Records
Source: Front Vet Sci. 2021 Dec 8;8:750183. doi: 10.3389/fvets.2021.750183 (PMC8692666; doi:10.3389/fvets.2021.750183)
Supplement: Supplementary file 1 [file Table_1.DOCX]

Supplementary Material

# Supplementary Table 1. Identification of the *ISCfe*1 sequence by PCR for the detection of *Campylobacter fetus* subsp. *venerealis* strains.

| Strain reference | Specie | Source | Laboratory | PCR Sequence target |
| --- | --- | --- | --- | --- |
|  |  |  |  | *ISCfe1* (Abril et al., 2007) |
| *Cfv* 1 | *C. fetus* subsp*. venerealis* | Bovine reference strain | RLC^a^ | + |
| *Cfv* 2 | *C. fetus* subsp*. venerealis* | Bovine reference strain | RLC^a^ | + |
| C2.2 | *C. fetus* subsp*. venerealis* | Bovine preputial scraping | LCV^b^ | + |
| C1.66 | *C. fetus* subsp*. venerealis* | Bovine preputial scraping | LCV^b^ | + |
| C1.84 | *C. fetus* subsp*. venerealis* | Bovine preputial scraping | LCV^b^ | + |
| C1.87 | *C. fetus* subsp*. venerealis* | Bovine preputial scraping | LCV^b^ | + |
| C1.88 | *C. fetus* subsp*. venerealis* | Bovine preputial scraping | LCV^b^ | + |
| C1.98 | *C. fetus* subsp*. venerealis* | Bovine preputial scraping | LCV^b^ | + |
| *Cff*1 | *C. fetus* subsp. *fetus* | Bovine reference strain | OIE^a^ | - |
| *Cff*2 | *C. fetus* subsp*. fetus* | Bovine reference strain | OIE^a^ | - |
| C1.54 | *C. fetus* subsp*. fetus* | Bovine preputial scraping | LCV^b^ | - |
| Slvt2 | *C. fetus* subsp*. fetus* | Bovine preputial scraping | SALUVET | - |
| 8228 | *C. sputorum* | Bovine preputial scraping | SALUVET | - |
| 8059 | *C. sputorum* | Bovine preputial scraping | SALUVET | - |
| 7908 | *C. sputorum* | Bovine preputial scraping | SALUVET | - |
| 6296 | *C. sputorum* | Bovine preputial scraping | SALUVET | - |
| 1281 | *C. sputorum* | Bovine preputial scraping | SALUVET | - |
| 1189 | *C. sputorum* | Bovine preputial scraping | SALUVET | - |
| 2967 | *Arcobacter cryaerophilus* | Bovine preputial scraping | SALUVET | - |
| 2609 | *Arcobacter cryaerophilus* | Bovine preputial scraping | SALUVET | - |
| *C. hyo hyo* | *C. hyointestinalis* subsp*. hyointestinalis* | Bovine reference strain | RLC^a^ | - |
| *C. hyo law* | *C. hyointestinalis* subsp*. lawsonii* | Bovine reference strain | RLC^a^ | - |

(+); PCR positive result, (-); PCR negative result.

*^a^* OIE Reference Laboratory for Campylobacteriosis at the Department of Infectious Diseases and Immunology, Faculty of Veterinary Medicine, Utrecht University.

*^b^* Central Veterinary Laboratory of Algete in Madrid.
